# Pharmacokinetic–Pharmacodynamic Simulation of Muscle Relaxation Antagonistic Conditions for Post-Operative Recurarization Prevention

**DOI:** 10.3390/jcm14062043

**Published:** 2025-03-17

**Authors:** Fumiyo Yasuma, Osamu Nagata, Yuka Matsuki, Kenji Shigemi

**Affiliations:** 1 Department of Anesthesiology and Reanimatology, University of Fukui, Fukui 910-1193, Japan; fyasuma@u-fukui.ac.jp (F.Y.); o-nagata@fa2.so-net.ne.jp (O.N.); 2 Department of Anesthesiology, Center Hospital of National Center for Global Health and Medicine, Tokyo 162-0052, Japan; 3 Department of Anesthesia, Touto Kasukabe Hospital, Saitama 344-0022, Japan; 4 Maizuru Municipal Hospital, Kyoto 624-0906, Japan; kshigemi@u-fukui.ac.jp

**Keywords:** pharmacokinetic–pharmacodynamic study, simulation model, rocuronium–sugammadex complex, muscle relaxant

## Abstract

**Background/Objectives:** No study has simulated rocuronium (Rb) effect-site concentrations (Ce_Rb) using real-time data—such as Rb concentrations, train-of-four (TOF) count (TOFC), and TOF ratio (TOFR)—under mechanical Rb administration. Therefore, we aimed to investigate post-operative recurarization and changes in the Ce_Rb after sugammadex (SGX) administration under conditions where Rb dosing was strictly administered using an automated delivery system for total intravenous anesthesia. **Methods:** This non-interventional, retrospective, observational study included 74 patients from an existing clinical trial who met the study criteria. Rb was automatically administered during surgery to maintain a TOFC of 1. SGX (2 mg/kg) was manually administered post-surgery, and the time until the TOFR reached ≥0.9 (if the time exceeded 3 min, 0.5 mg/kg SGX was added every minute). The results were analyzed using a pharmacokinetic (PK)–pharmacodynamic (PD) simulation model of the Rb-SGX complex. **Results:** The average total dose of administered SGX was 2.2 ± 0.4 mg/kg (mean ± standard deviation). The time from SGX administration till the TOFR reached ≥0.9 was 2.9 ± 1.1 min. Furthermore, Ce_Rb at recovery (Ce_r) was 0.3 ± 0.2 μg/mL. Notably, no cases showed post-operative recurarization within 24 h of surgery. PK–PD model simulations revealed that Ce_Rb increased again after reaching the lowest Ce_Rb in 72 cases, although no increase was recorded beyond Ce_r, suggesting no numerical risk of recurarization. **Conclusions:** Our results show that if TOFC of 1 is strictly maintained intraoperatively and SGX is administered till the TOFR reaches ≥0.9, post-operative recurarization does not occur.

## 1. Introduction

The muscle relaxant antagonist, sugammadex (sodium) (SGX), is widely used as an antagonist of the muscle relaxant, rocuronium bromide (Rb). However, problems can arise from residual muscle relaxation and recurring muscle relaxation (also known as recurarization) in cases of insufficient antagonism. Residual or recurring muscle recurarization after general anesthesia increases postoperative respiratory complications, prolongs the length of hospital stay owing to delayed postoperative recovery and other complications, and increases medical costs. Thus, the prevention of postoperative residual muscle relaxation or recurarization is crucial for achieving a better postoperative course. To avoid these problems, the importance of monitoring muscle relaxation has been highlighted. Using a pharmacokinetic (PK)–pharmacodynamic (PD) model that includes the response to two drugs, a muscle relaxant and a muscle relaxant antagonist, it is possible to predict the pharmacokinetics of free Rb at the site of action after SGX administration. Using this free Rb concentration, it is predicted that recurarization will occur during the period when the concentration of Rb has exceeded the concentration at the time of recovery, when the muscle relaxation effect has disappeared, that is, when the Train-of-Four (TOF) Ratio (TOFR) is ≥0.9.

We examined the concentration of Rb during muscle relaxation recovery and clinical symptoms using a pharmacokinetic simulation model proposed by Kleijn et al. [1]. The results clinically and numerically confirmed that recurarization does not occur if the recommended dose of SGX is administered within the measured TOFR [2].

However, since the administration of muscle relaxants is at the discretion of the anesthesiologist, analysis of the site of action of Rb concentration (Ce_Rb) after SGX administration with strict control of Rb dose and TOF values was not possible due to the variability of muscle relaxation states.

Recently, automated intravenous anesthesia systems have shown notable development and application potential in clinical settings, and our research has reported that these systems are on a par to maintain the stable drug effects with the level of drug administration adjustment by anesthesiologists [3].

Herein, we aimed to investigate the risk of postoperative recurarization, along with the changes in Ce_Rb after SGX administration under a strictly controlled muscle relaxation state. We used the data obtained from the clinical study jRCTs052200118 (ROP-CT) to perform PK–PD simulations of the Rb–SGX complex.

## 2. Materials and Methods

### 2.1. Ethics Approval

This non-interventional, retrospective, observational study included patients from a clinical study (jRCTs052200118, hereafter referred to as ROP-CT) that investigated the balance/need for propofol and remifentanil during combined epidural anesthesia/neuroblock using an automatic anesthetic drug administration system. The inclusion and exclusion criteria for the ROP-CT were as follows:

Inclusion criteria: patients who meet all of the following criteria:

(1) Males and females aged 20 years at the time of obtaining consent.
(2) Patients with the American Society of Anesthesiologists–physical status classification system (ASA-PS) of 1–3 who will receive general anesthesia using propofol, remifentanil, and rocuronium.

Exclusion criteria: patients who fall under any of the following conditions:

(1) Patients with a history of hypersensitivity to propofol, remifentanil, rocuronium, or sugammadex.
(2) Patients who could not have the Bispectal Index (BIS) sensor attached during surgery.
(3) Patients who could not receive additional rocuronium after the initial single dose.
(4) Patients who cannot undergo noninvasive blood pressure measurements during surgery.
(5) Patients who underwent hypothermic surgery.
(6) Patients undergoing cardiovascular surgery.
(7) Pregnant, breastfeeding, or planning to become pregnant.
(8) Participation in another study within 12 weeks prior to obtaining consent or planning to participate in another study during the study period.
(9) Patients whom the principal investigator or sub-investigator deems unsuitable for inclusion in the study.

### 2.2. Anesthesia Procedures

The automated delivery system for total intravenous anesthesia (TIVA) is Software as a Medical Device (SaMD), which controls a group of syringe pumps that deliver three intravenous anesthetics (propofol, remifentanil, and rocuronium) based on information automatically transferred from a biometric monitor. The device used in this study is a medical device equipped with a software program that uses a closed-loop control system to calculate the optimal dose of a drug based on PK analysis of the biometric information of the subject and automatic adjustment of the results [3].

For the induction of general anesthesia, start by administering remifentanil at a dose of 0.5 μg/kg/mL, based on the ideal body weight. When the remifentanil effect-site concentration reaches ≥5 ng/mL, the device will administer a bolus dose of propofol at 1 mg/kg, followed by continuous administration at 10 mg/kg/min. The BIS value will be set to 55 or less, or the device will automatically switch to automatic control mode 3 min after the start of continuous propofol administration. Rocuronium was administered after confirming that the patient had fallen asleep and after calibrating the TOF monitor. The details of the rocuronium administration method are described in the following paragraph. During surgery, the three drugs were administered and adjusted using an automatic administration control system.

Muscle relaxation was monitored using an electromyographic muscle relaxation module (AF-201P, Nihon Kohden Corporation, Tokyo, Japan). The stimulating electrode was placed on the ulnar nerve, and the guiding electrode was affixed to the abductor muscle and proximate tendons of the fifth digit, and TOF stimulations were performed at 12 s intervals. Initially, a 0.6 mg/kg (ideal body weight) Rb dose was administered for anesthesia induction. If the TOF count (TOFC) does not reach 0, 1 min after the initial dose, an additional dose of 0.3 mg/kg should be administered. Subsequently, it was automatically adjusted to maintain a TOFC of 1 during surgery. According to the specifications of the equipment, if TOFC = 0 is not reached after 3 min of an additional Rb administration, Rb dosing is automatically started at a rate of 7 μg/kg/min after 3 min, and auto-adjustment is started after 12 s. If TOFC = 0 is not reached after 12 min of continuous dosing, or if a TOFC of ≥3 continues for 5 min, it is considered abnormal, and a system warning is issued. Rb administration was stopped after surgery, and a responsible anesthetist recorded the timing and dose of manually administered SGX (2 mg/kg; actual body weight) on the anesthesia chart. The time from SGX administration till the TOFR reached ≥0.9 was measured using a stopwatch. If the recorded time exceeded 3 min, an additional SGX dose of 0.5 mg/kg was administered every minute until the TOFR reached ≥0.9.

### 2.3. Analysis of Patient Demographics

This study was based on the ROP-CT data. Age, sex, height, weight, and serum creatinine (Cr) levels were denoted as the patient data, and the muscle relaxation status (TOFC/TOFR values), Rb dosage, and SGX dosage were denoted as the intraoperative data. Furthermore, the occurrence of recurarization within 24 h after operation was investigated based on adverse event items in the ROP-CT.

The ROP-CT was used to evaluate the balance between sedatives and analgesics. Therefore, although the exclusion criteria did not explicitly state that patients with hepatic/renal failure were excluded, patients with complications that significantly affected drug metabolism were excluded at the discretion of the researchers.

The exclusion criteria for this study were: (1) patients with a body mass index (BMI) values outside the range of 18–26 kg/m^2^, (2) patients whose TOFR ≥ 0.9 could not be confirmed by the ROP-CT data, (3) patients with unstable muscle relaxation monitoring data during the procedure, and (4) patients who were not administered with SGX as per the protocol.

### 2.4. PK–PD Model of the Rb–SGX Complex

Our development research adopted the model devised by Wierda et al. [4] as the PK model for Rb administration. Rb was administered according to the ideal body weight. The system recorded drug doses in absolute μg/min every 12 s, as comma-separated value files on a general-purpose computer. SGX was administered manually, and the time to muscle relaxation recovery (TOFR > 0.9) was measured and recorded. The Kleijn model [1] was used to recalculate the blood concentration of Rb (C_Rb) and the concentration at the effect site (Ce_Rb) from the Rb doses obtained from the dosing records. The Kleijn model simulations were based on the ideal body weight. Furthermore, C_Rb/Ce_Rb/Ce_r was calculated via the Kleijn model using the actual SGX dose and TOFR/TOFC data recorded in the system.

Herein, the PK–PD model of the Rb–SGX complex, as proposed by Kleijn et al. [1], was used. This model is formed using a two-compartment model, which is based on data obtained from eight clinical studies including patients of different age, race, and renal function parameters. Although creatinine clearance (CCr) is used in the model, the estimated CCr (eCCr) determined using the Cockcroft–Gault equation was analyzed in this study. In the Kleijn model, Asian and non-Asian populations were listed as covariates, and in this study, all the calculations were performed based on the parameters of Asian populations.

### 2.5. Statistical Analysis

**Figure 1:**
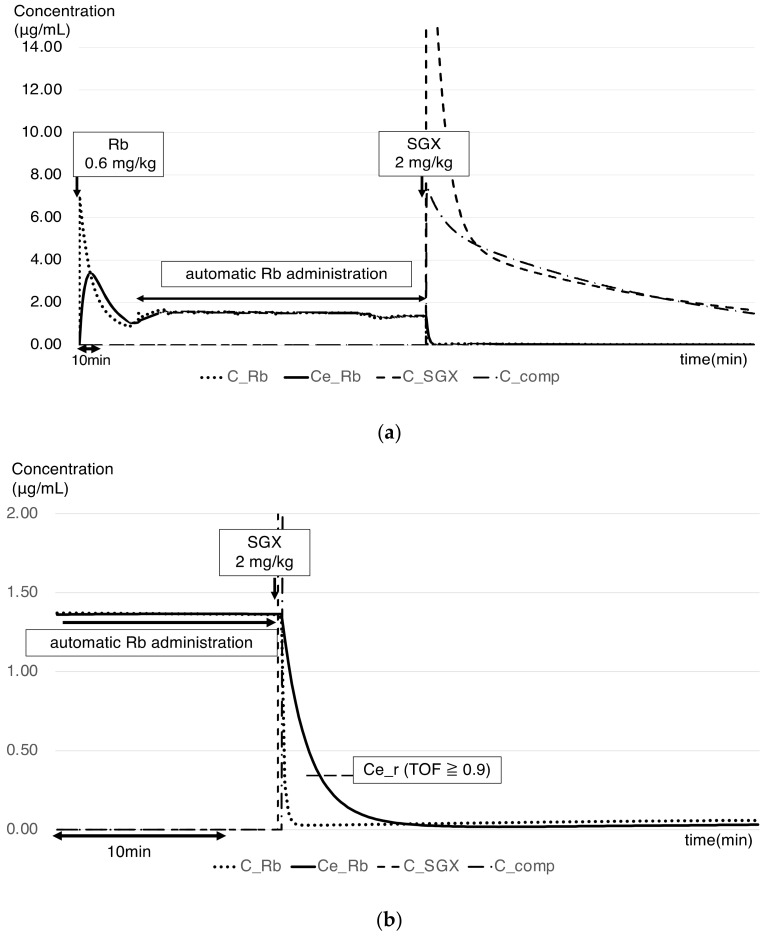
Graphs of the pharmacokinetic model proposed by Kleijn et al. [1] (**a**) Blood and effect-site concentrations of administered Rb and SGX, as determined using the pharmacokinetic model proposed by Kleijn et al. [1] (**b**) Changes in blood concentrations and effect-site concentrations after SGX administration (expansion of (**a**)). C_Rb, rocuronium blood concentration; Ce_Rb, rocuronium effect-site concentration; Ce_r, concentration of rocuronium effect site during recovery of muscle relaxation (TOFR ≥ 0.9); C_SGX, sugammadex blood concentration; C_comp, rocuronium–sugammadex complex blood concentration.

PK simulation was performed using the Rb–SGX complex (PK–PD model proposed by Kleijn et al.) [1]. The blood concentrations of Rb/SGX/complex and Ce_Rb were plotted graphically based on the Rb and SGX dosing history (Figure 1a).

The primary endpoint was denoted as the presence or absence of a risk of recurarization. Ce_Rb was calculated as Ce_r at a TOFR of ≥0.9 (Figure 1b), and a graph showing the transition of Ce_Rb after reaching Ce_r was plotted to determine the time at which Ce_Rb exceeded Ce_r (the time where the risk of recurarization was recorded).

For the secondary endpoints, the correlations among age, renal function, and intraoperative targets Ce_Rb and Ce_r were examined. The correlation coefficient is expressed by r; 0.7 ≤ |r| ≤ 1 indicates a strong correlation, 0.4 ≤ |r| ≤ 0.7 indicates a correlation, 0.2 ≤ |r| ≤ 0.4 indicates a weak correlation, and 0 ≤ |r| ≤ 0.2 indicates no correlation. A significance level of less than 5% was considered significant in the statistical analysis.

In a subgroup analysis, we compared the patient parameters to determine whether there were any differences between the groups that required additional SGX administration (Group A) and those that did not (Group NA). Two-sided *p*-values were calculated using the Student’s *t*-test. The results are shown as mean ± standard deviation values.

## 3. Results

**Figure 2:**
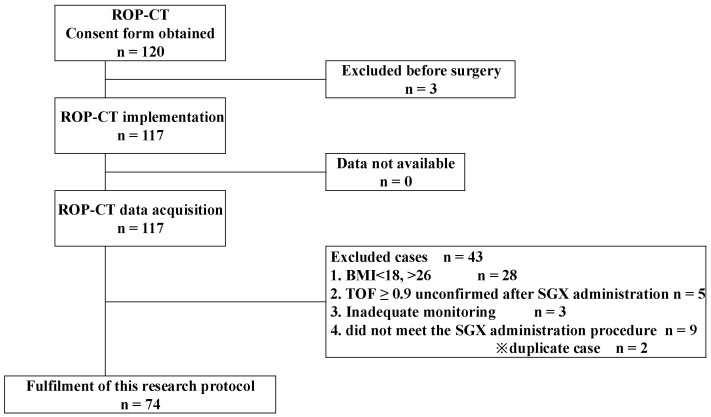
Flowchart showing patient inclusion. ROP-CT, clinical study jRCTs052200118.

Of the 117 patients enrolled in the ROP-CT, 74 patients who met the inclusion criteria were included in the present study (Figure 2). The patient background data are shown in Table 1. Only one patient did not reach TOFC = 0 after the additional dose; therefore, continuous rocuronium administration was initiated in accordance with the device specifications. The average total SGX dose administered was 2.2 ± 0.4 mg/kg, and the average time from SGX administration to complete recovery of muscle relaxation was 2.9 ± 1.1 min. Moreover, 24 patients needed additional SGX. Of these, eight patients required more than one additional dose of SGX (two times [total SGX dose, 3 mg/kg] for seven patients, three doses [3.5 mg/kg] for one patient). Notably, no patient presented with clinical recurarization within 24 h postoperatively.

**Figure 3:**
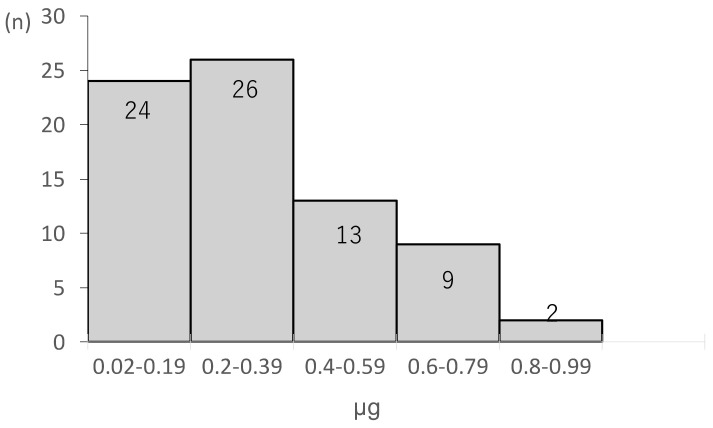
Ce_r values calculated using the Kleijn model. Ce_r, concentration of rocuronium effect site during recovery of muscle relaxation.

**Figure 4:**
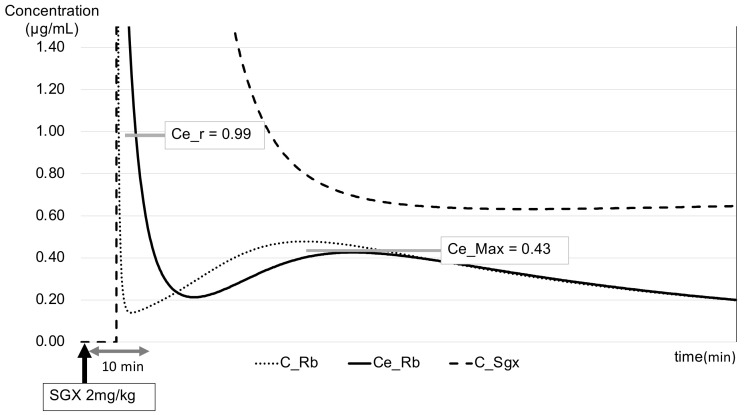
Graph of blood and effect-site concentrations after SGX administration (cases with Ce_r = 0.99 μg/mL and Ce_Max = 0.43 μg/mL).

The range of the target Ce_Rb under TOFC = 1 was 0.29–3.0 μg/mL. The results showed that Ce_Rb decreased after SGX administration, and Ce_Rb (also referred to as Ce_r at this point) was 0.3 ± 0.2 μg/mL (range: 0.02–0.99) at a TOFR of >0.9 (Figure 3). A further decrease in Ce_Rb was noted, and it reached its lowest value after 6.9 ± 2.4 min. In 72 cases, Ce_Rb showed a gradual increase after reaching its lowest value, and the highest value of Ce_Rb after the second increase (namely Ce_max) was found to be 0.05 ± 0.05 μg/mL (range: 0.01–0.43) after 27.4 ± 7.0 min from the timepoint of Ce_r (Figure 4). Herein, no re-increase was observed in Ce_Rb above Ce_r after SGX administration in any of the cases.

**Figure 5:**
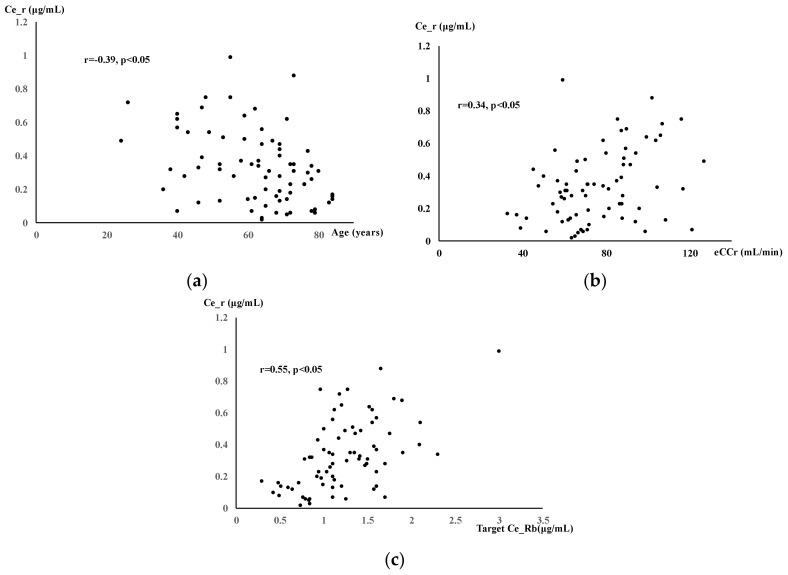
Correlation between rocuronium (Rb) concentration at recovery Ce_r. (**a**) Age and Ce_r; Ce_r showed a decreasing trend with age. (**b**) eCCr and Ce_r; a weak correlation was observed between eCCr and Ce_r. (**c**) Intraoperative target Ce_Rb (train-of-four count [TOFC] = 1) and Ce_r; a weak positive correlation was observed between Rb effect-site concentration and Ce_r to obtain TOFC = 1. eCCr, estimated creatinine clearance calculated using the Cockcroft–Gault formula.

Regarding the secondary endpoints, the correlation coefficients between age, eCCr, and the intraoperative target Ce_Rb and Ce_r were r = –0.39, 0.34, and 0.55, respectively, with only the target Ce_Rb showing a correlation (Figure 5), while the others showed weak correlations.

A subgroup analysis was conducted on 24 patients (Group A) who required additional SGX administration and the others (Group NA). The results of the comparative study of age, renal function, actual body weight, intraoperative rocuronium dose, and Ce_r are provided in Table 2. No significant differences exist between the two groups in terms of age, actual body weight, eCCr, Rb dose per operative time, or the intraoperative target Ce_Rb levels. Ce_r was significantly lower in the SGX group.

## 4. Discussion

In this study, the Rb–SGX PK–PD model was used to simulate the changes in muscle relaxant effect-site concentrations after administering the antagonist. Furthermore, the effect-site concentrations during the recovery of muscle relaxation and the risk of recurarization were investigated. To the best of our knowledge, this is the first study to simulate Rb effect-site concentrations using real-time data (such as Rb concentrations, TOFC, and TOFR) under mechanical Rb administration. However, SGX was administered manually.

After administering SGX, both the free Rb and SGX-encapsulated Rb are found in the blood, and they cannot be measured separately [1,5]. This results in an increase in the numerical Rb concentration following SGX administration compared with that before administration. This result is also consistent with the findings shown in our previous study [6]. The quantification of Rb concentrations during the recovery of muscle relaxation is difficult because SGX and Rb blood concentrations after SGX administration exhibit dynamic changes in a short period, in the order of seconds and minutes. Therefore, investigating the concentration of free Rb during the recovery of muscle relaxation using a simulation model is a potential approach.

The results of this study indicate that if the effect-site concentration of the muscle relaxant that results in a TOFR of ≥0.9 is not obtained, this simulation cannot be performed. This finding suggests that we could not predict the necessary and sufficient amounts of muscle-relaxant antagonists before administration.

The value of Ce_r varies significantly from person to person. Patients with high Ce_r may be at a high risk of residual muscle relaxation; however, it may be necessary to consider the intraoperative target Ce_Rb. The results of this study show a correlation between the target Ce_Rb and Ce_r. If the TOFR ≥ 0.9 can be confirmed after SGX administration, the risk of residual muscle relaxation will be low regardless of the Ce_r value. Of course, it is important to confirm that the patient is stable with a TOFR of ≥0.9 and that they have recovered from the muscle relaxation state based on clinical findings before extubation. The results for the target Ce_Rb show a large degree of individual variation. In a study investigating the Rb infusion rate and blood concentration required to obtain T1 3–10% under TIVA, the predicted concentration was 1.7 ± 0.5 µg/mL on average, with a minimum of 0.8 and a maximum of 2.9, and the average blood concentration was 1.4 ± 0.4 µg/mL [7]. This shows that there were large individual differences in the amount of Rb required to achieve sufficient muscle relaxation during surgery.

This study differs from our previous study [2] in the following aspects: (1) the use of ROP, (2) whether the muscle relaxation monitor was an accelerometer or myoelectric type, and (3) the method of SGX administration. Regarding the monitoring of muscle relaxation, no significant differences in the measured values were reported between the acceleration-type and electromyography-type monitors [8,9]. Herein, if the TOFR did not reach ≥0.9 at 3 min after SGX administration, an additional 0.5 mg/kg SGX was administered.

We examined whether there were any differences in the patient parameters between the 50 patients who achieved a TOFR of ≥0.9 within 3 min of the initial 2 mg/kg dose and the 24 patients who required additional SGX administration. No significant differences were observed in regard to age, renal function, or intraoperative Rb dose; however, Ce_r was significantly lower in group A. As additional SGX accelerated the decrease in Ce_Rb, it is reasonable to assume that Ce_r was lower in group A. Initially, we expected that group A would use more Rb; however, the results showed no difference. According to the ROP-CT protocol, the muscle relaxant is stopped simultaneously with the sedative and analgesic agents; thus, automatic Rb administration is performed to achieve TOFC = 1 immediately before SGX administration. It is possible that the speed of administration before RB was discontinued affected the amount of SGX required.

The present consensus recommends the administration of 4 mg/kg SGX for deep muscle relaxation (post-tetanic count; PTC1-2) [10] and 2 mg/kg SGX for shallow muscle relaxation (TOFC = 2) [11]. These recommendations are based on the measurements of the time to recover to TOFR = 0.9 after SGX administration in patients maintained under anesthesia using sevoflurane in each muscle relaxation state; the results showed a dose-dependent reduction in recovery time [10,11]. However, the SGX dosage requirement at the TOFC = 1 state and in the even shallower muscle relaxation states recovering to TOFR appearance remains unclear. By contrast, a study states that administering 0.5 or 1 mg/kg SGX at TOFC = 2 or PTC = 1–3 in adult patients with the ASA-PS 1 or 2, no additional SGX is required if the TOFR reaches ≥0.9 at 3 min after initial SGX administration [12]. Herein, SGX was administered rigorously, maintaining the TOFC at 1 during the surgery with TIVA. To confirm that the recovery of muscle relaxation occurs at a TOFR of ≥0.9, 2 mg/kg SGX was initially administered, which was followed by an additional 0.5 mg/kg SGX administration if no recovery was observed after 3 min. Using this method, safety was ensured as complete antagonism was maintained throughout the period.

Kleijn et al. performed clinical dosage simulations and showed that the predicted recovery time was always <2 min in healthy subjects, along with the limited effects of age, renal function, and sevoflurane use [1]. In our study, the tendency of Ce_r to be lower in the elderly is consistent with the findings of previous studies reporting a slower recovery time from muscle relaxation with increasing age owing to factors such as reduced cardiac output and changes in reduced muscle blood flow at the neuromuscular junction [13,14,15,16]. SGX is primarily excreted via the kidneys; thus, many studies have explored its role in patients with impaired renal function [17,18,19,20]. Notably, the time to recover to a TOFR of ≥0.9 in patients with impaired renal function has been reported to not significantly differ compared with that in healthy subjects [17]. Some studies have reported significantly longer TOFR recovery times in patients undergoing dialysis than those in healthy subjects, but the difference in the recovery rate was insufficient to be clinically significant [20]. In patients with liver failure, the distribution volume increases, and accumulation in the body increases; as a result, there is a possibility that the excretion of rocuronium will be delayed and the duration of action will be prolonged [21]. This study did not include patients with liver failure or those undergoing hemodialysis.

In a series of automated delivery systems for TIVA, all Rb doses were determined using ideal body weight calculations. When Rb is administered on an actual body weight basis, the duration of action is markedly prolonged in patients with obesity compared to that in normal-weight patients. This is due to the increase in the amount of fat in the body weight, and because Rb is not easily transferred to fat, so it is a relative overdose when administered on a body weight basis [22]. Moreover, it would be preferable to administer the drug in terms of fat-free mass (FFM). We did not measure the body fat percentage of all the patients at our facility; therefore, we substituted the ideal body weight. In the future, we would like to conduct a study on the intraoperative administration of Rb in terms of FFM, including patients with a BMI > 26 kg/m^2^.

This study had some limitations. (1) Patient issues: Patients with diabetes and peripheral neuropathy were not excluded in this study, and the presence or absence of antiepileptic medication was not considered. It has been reported that the time it takes for the TOFR to reach 0.9 is longer for diabetic patients than for other patients [23], and that taking antiepileptic drugs shortens the effect of rocuronium [24]. Thus, these factors may have introduced a confounding bias. The applicability of the results of this study to patients with liver or renal failure is a topic for future research. In addition, this study included patients who had undergone epidural anesthesia or a peripheral nerve block. The effects of local anesthetics were not considered in this study. Moreover, this study included patients equipped with tourniquets, which may have affected the blood concentration or effect-site concentration of muscle relaxants. (2) Effects of drug administration methods. Owing to the manual administration of SGX, differences may be observed between the time recorded as the dosing time and the time that SGX reached the body. The positional relationship between the blood pressure and muscle relaxation monitors was unclear. Finally, in clinical practice, the state of muscle relaxation may deviate from TOFC = 1. If a patient maintains a deeper state of muscle relaxation during surgery, it is necessary to further investigate whether these results can be applied.

This study was conducted before the publication of the Good Clinical Practice III guidelines for PD studies of neuromuscular blocking agents [25], so in future studies, we would like to determine the amount of SGX required in accordance with these guidelines.

## 5. Conclusions

The findings of this study showed that under the strict regulation of the Rb dose (TOFC = 1), the administration of SGX—until a TOFR of ≥0.9 was reached—did not cause clinical recurarization. Additionally, PK–PD simulations revealed no risk of recurarization. The monitoring of muscle relaxation and PK–PD simulations confirmed the safety of the muscle relaxation antagonism method employing robotic anesthesia systems. Overall, this study provides a theoretical basis for further investigations and the wide use of the muscle relaxation antagonism method in clinical settings.

## Figures and Tables

**Table 1:** Patient background and clinical data.

Background Factors		Values
Sex	Male/Female	25:49
Age (years)	Mean ± SD	62.2 ± 14.0
Height (cm)	Mean ± SD	159.0 ± 7.8
Weight (kg)	Mean ± SD	56.3 ± 8.1
BMI (kg/m^2^)	Mean ± SD	22.3 ± 2.2
ASA-PS	I:II:III	21:51:2
eCCr (mL/min)	Mean ± SD	75.9 ± 20.6
Operation time (h)	Mean ± SD	2.5 ± 1.0
Rb dose (mg/operation time (h))	Mean ± SD	43.8 ± 16.1
Total SGX dose (mg/kg)	Mean ± SD	2.2 ± 0.4
Recovery time (min) (from SGX administration till the TOFR reached ≥0.9)	Mean ± SD	2.9 ± 1.1

ASA-PS: American Society of Anesthesiologists–physical status; BMI: body mass index; eCCr: estimated creatinine clearance calculated using the Cockcroft–Gault formula; SD: standard deviation; Rb: rocuronium; SGX: sugammadex; TOFR: Train-of-four ratio.

**Table 2:** Comparison of Group A and Group NA.

Factors	Group NA n = 50	Group A n = 24	*p-*Value
Age (years)	60.1 ± 15.1	66.5 ± 9.9	0.067
Actual body weight (kg)	56.8 ± 8.3	55.6 ± 7.8	0.557
eCCr (mL/min)	77.4 ± 20.7	72.8 ± 19.9	0.380
Rb dose (mg/h)	44.2 ± 17.6	42.1 ± 12.5	0.602
Target Ce_Rb (μg/mL)	1.25 ± 0.48	1.18 ± 0.41	0.531
Ce_r (μg/mL)	0.42 ± 0.2	0.16 ± 0.11	<0.05

Values are expressed as mean ± standard deviation. Rb dose, total Rb dose (mg), and operative time (h).

## Data Availability

The data that support the findings of this study are available from the corresponding author upon reasonable request.

## Abbreviations

The following abbreviations are used in this manuscript:

ASA-PS	American Society of Anesthesiologists–physical status
BIS	Bispectal Index
GCRP	Good clinical research practice
SaMD	Software as a Medical Device
TIVA	Total intravenous anesthesia
TOF	Train-of-four
TOFC	Train-of-four count
TOFR	Train-of-four ratio
